# Detection and serotyping of pneumococci in community acquired pneumonia patients without culture using blood and urine samples

**DOI:** 10.1186/s12879-015-0788-0

**Published:** 2015-02-13

**Authors:** Karin Elberse, Suzan van Mens, Amelieke J Cremers, Sabine CA Meijvis, Bart Vlaminckx, Marien I de Jonge, Jacques F Meis, Cornelis Blauwendraat, Ingrid van de Pol, Leo M Schouls

**Affiliations:** Centre for Infectious Disease Control Netherlands, National Institute for Public Health and the Environment (RIVM), Laboratory for Infectious Diseases and Perinatal Screening, Antonie van Leeuwenhoeklaan 9, P.O.Box 1, 3720 BA Bilthoven, The Netherlands; Departments of Medical Microbiology & Immunology, Sint Antonius Hospital Nieuwegein, P.O box 2500, 3430 EM Nieuwegein, The Netherlands; Department of Internal medicine, Sint Antonius Hospital Nieuwegein, P.O box 2500, 3430 EM Nieuwegein, The Netherlands; Laboratory of Pediatric Infectious Diseases, Department of Pediatrics, Radboud University Medical Center, Geert Grooteplein-Zuid 10, 6525 GA Nijmegen, The Netherlands; Laboratory of Medical Immunology, Department of Laboratory Medicine, Radboud University Medical Center, Geert Grooteplein-Zuid 10, 6525 GA Nijmegen, The Netherlands; Department of Medical Microbiology, Radboud University Medical Center, Geert Grooteplein-Zuid 10, 6525 GA Nijmegen, The Netherlands; Department of Medical Microbiology and Infectious Diseases, Canisius Wilhelmina Hospital, Weg door Jonkerbos 100, 6532 SZ Nijmegen, The Netherlands

**Keywords:** *Streptococcus pneumoniae*, Pneumococcus, Community acquired pneumonia, Detection, Serotype, Blood, Urine

## Abstract

**Background:**

Treatment of community acquired pneumonia (CAP) patients with antibiotics before laboratory-confirmed diagnosis leads to loss of knowledge on the causative bacterial pathogen. Therefore, an increasing number of pneumococcal infections is identified using non-culture based techniques. However, methods for serotyping directly on the clinical specimen remain scarce. Here we present three approaches for detection and serotyping of pneumococci using samples from patients with CAP.

**Methods:**

The first approach is quantitative PCR (qPCR) analysis on blood samples (n = 211) followed by capsular sequence typing (CST) to identify the serotype. The second approach, a urinary antigen assay (n = 223), designated as inhibition multiplex immunoassay (IMIA), is based on Luminex technology targeting 14 serotypes. The third approach is a multiplex immunoassay (MIA) (n = 171) also based on Luminex technology which detects serologic antibody responses against 14 serotypes. The three alternative assays were performed on samples obtained from 309 adult hospitalized CAP patients in 2007–2010 and the results were compared with those obtained from conventional laboratory methods to detect pneumococcal CAP, i.e. blood cultures, sputum cultures and BinaxNOW® urinary antigen tests.

**Results:**

Using qPCR, MIA and IMIA, we were able to detect the pneumococcus in samples of 56% more patients compared to conventional methods. Furthermore, we were able to assign a serotype to the infecting pneumococcus from samples of 25% of all CAP patients, using any of the three serotyping methods (CST, IMIA and MIA).

**Conclusion:**

This study indicates the usefulness of additional molecular methods to conventional laboratory methods for the detection of pneumococcal pneumonia. Direct detection and subsequent serotyping on clinical samples will improve the accuracy of pneumococcal surveillance to monitor vaccine effectiveness.

## Background

*Streptococcus pneumoniae* is a major human pathogen causing considerable morbidity and mortality throughout the world. It is considered the main cause of community acquired pneumonia (CAP) [1,2], although there is little consensus in the literature on the prevalence of pneumococci in CAP, it ranges from 10 to 48% in hospitalized patients [1]. Differences are observed between countries and health care settings, but also differences in methods used to identify the causative agent contribute to the diverse numbers of prevalence [3,4]. The lack of sensitive methods to identify the pathogen adds to the problem [5-10]. Furthermore, patients are often treated with antibiotics before the collection of specimens for laboratory diagnosis, making the identification of the causative agent more problematic.

Standard microbiology assays to detect the pneumococcus as the causative agent of CAP are culture from sputum and/or blood, sputum gram stain and the BinaxNow *S. pneumoniae* test. The BinaxNow test is a rapid antigen test on urine or cerebrospinal fluid, which detects pneumococcal cell wall polysaccharides (CWPS). Specificity and sensitivity are high in adults for the diagnosis of pneumococcal CAP compared with conventional methods [6,11,12]. In children, specificity is much lower due to high carriage rates [5,13]. Developed assays for detection of pneumococcal DNA in blood samples include PCR and quantitative PCR (qPCR) [14-16]. Recently, a few studies described the use of PCR to identify the serotype directly from pneumococcal DNA present in blood [17-19]. These PCRs contain a myriad of primers and/or probes, using single- or multiplex reactions, making the method complex.

Currently, over 90 pneumococcal serotypes have been described and roughly a quarter of these serotypes are responsible for the majority of cases of invasive pneumococcal disease (IPD) [20-24]. Serotyping of pneumococci is essential in monitoring the effects of nationwide vaccine introduction. The classical technique for serotyping is the Quellung reaction, based on reactivity of the capsular polysaccharides with specific antisera [7,25]. To identify a pneumococcal serotype using the Quellung reaction, a cultured isolate must be present. The Quellung reaction is time-consuming and expensive because a whole collection of antisera is needed for the identification of all serotypes. Many alternative serotyping methods have been developed [17-19,26]. One of these is the Capsular Sequence Typing (CST) [27,28], a method based on the sequence obtained from a single PCR product of the capsular gene *wzh*.

Measurement of serotype-specific antibodies has been time-consuming due to the lack of multiplexing ability of the Enzyme-linked immuno sorbent assay (ELISA). With the introduction of the Luminex platform, simultaneous measurement of IgG concentrations directed against a large number of different capsular polysaccharides in a single assay became possible. This method was first described by Pickering et al. for pneumococci [29] and later adjusted and optimized by others [30,31]. In a sandwich or inhibition version of the first described pneumococcal Luminex assay, also detection of pneumococcal polysaccharides in urine samples appeared to be possible [10,32,33].

In this study, we describe three methods to assess the pneumococcal serotype directly from urine and blood. We developed an assay to conduct the serotyping by CST directly on pneumococcal DNA isolated from blood samples by a rapid extraction method. Furthermore, we describe an inhibition assay approach to detect pneumococcal capsular polysaccharides of 14 different serotypes in urine. The serotypes covered by this assay represent the serotypes in the 13-valent pneumococcal vaccine and serotype 8 which is frequently found in IPD patients. The third assay is previously described and detects the causative pneumococcus by a positive antibody response [9]. The methods are used to detect and serotype pneumococci in CAP patients and the results from these assays are compared with conventional microbiology assays.

## Methods

### Clinical samples

Blood and urine samples were obtained from hospitalized CAP patients aged 18 to 100 years, who participated in a trial assessing the effect of dexamethasone use in CAP. This study was carried out in the St. Antonius Hospital, Nieuwegein, The Netherlands and is described in detail elsewhere [34]. Mean age of the enrolled patients was 63 years old and approximately 27% of the patients started antibiotic treatment before enrolment. Samples were collected from November 2007 to September 2010 and conventional laboratory methods to identify the causative agent of CAP were performed. Additionally, ethylenediamine-tetra-acetic acid (EDTA) blood samples for molecular testing were obtained on the day of admission and sent to the National Institute for Public Health and the Environment (RIVM) within 1 day after the blood was drawn. Urine samples for IMIA antigen testing were collected on the day of admission, sent to the RIVM and stored at −20°C until use. Serum samples for the detection of antibody responses by MIA were obtained on the day of admission and at day 30 after admission and stored at −80°C. Eligible patients provided written informed consent and the study was approved by the institutional Medical Ethics Committee of the St. Antonius Hospital. This study was registered with ClinicalTrials.gov, number NCT00471640. For comparison of the CST, serum samples and blood culture isolates were collected from adults with pneumococcal bacteremia hospitalized in a different institution between December 2008 and June 2013.

### Conventional laboratory methods

Diagnostic tests were performed on sputum, blood and urine samples at the day of admission to identify the causative agent of CAP as previously described [34]. Briefly, sputum samples were Gram stained and cultured and at least two blood cultures were performed (BacT/Alert; bioMérieux, Marcy l’Etoile, France). Urine samples were used for detection of *S. pneumoniae* antigen (BinaxNOW *S. pneumoniae,* Inverness Medical). The serotypes of the pneumococcal isolates used for comparison of the CST were determined by multiplex PCR analysis of the *cps* locus as described by Pai *et al.* [26] or by Quellung reaction [25].

### Molecular blood tests

#### Sample preparation

EDTA blood sample tubes were centrifuged for 10 minutes at 400×g. A milliliter of plasma was transferred to a clean tube and was centrifuged for 10 minutes at 14000xg. After transferring the plasma, the pellet was centrifuged again for one minute at 14000xg and residual plasma was discarded. From the pellet the pneumococcal DNA was extracted using the Extract-N-Amp Plant PCR Kit (Sigma-Aldrich, St. Louis, MO, USA). In detail, 25 μl of extraction buffer was added to the pellet and incubated at 95°C for 10 minutes. Thereafter, 25 μl of dilution buffer was added and the material was ready for use in the qPCR. For use in the CST, the samples were purified using a Sephadex column (G-50, GE Healthcare, Chalfont St. Giles, UK), according to manufacturer’s protocol.

From 200 μl serum samples used for comparison of the CST, total DNA was extracted using the MagNA Pure 96 Pathogen Universal 200 Kit (Roche Diagnostics, Indianapolis, IN, USA) and eluted in 100 μl elution buffer.

#### Detection qPCR

The quantitative PCR was based on two target genes, *ply* and *lytA*, as described before [8,35]. Sequences of the primers and probes are provided in Table 1. An internal control was added to detect inhibitors of amplification and contained a primer site for the *lytA* forward primer and a primer site for the *ply* reverse primer. To amplify the two gene segments, 10 μl PCR mixtures containing QuantiTect Multiplex PCR NoROX Kit, (Qiagen, Hilden, Germany), 0.6 μM of each primer, 0.3 μM of each probe, 1000 copies of internal control and 2 μl of the extracted DNA were used. The PCR reaction was as follows: 2 min 50°C, 15 min 95°C and 40 cycles of 15 sec 95°C and 1 min 65°C in a LightCycler 480 (Roche, Mannheim, Germany). The standard curve consisted of PCR products of *ply* and *lytA* gene segments which were purified using Qiaquick PCR Purification Kit (Qiagen) and quantified using a spectrophotometer (Nanodrop ND-1000, Thermo Scientific, Wilmington, DE, USA). The PCR was considered positive for pneumococci if at least one of the duplicate samples yielded a fluorescent signal for *lytA*.

**Table 1:** **Oligonucleotide primers, probes and internal control sequences**

**Assay**	**Name**	**Sequence**
qPCR	Sp-ply-531 F^1^	AGCGATAGCTTTCTCCAAGTGG
	Sp-ply-583R^1^	CTTAGCCAACAAATCGTTTACCG
	Sp-lytA-306 F^1^	ACGCAATCTAGCAGATGAAGC
	Sp-lytA-386R^1^	TGTTTGGTTGGTTATTCGTGC
	Sp-lytA-CDCF^2^	ACGCAATCTAGCAGATGAAGCA
	Sp-lytA-CDCR^2^	TCGTGCGTTTTAATTCCAGCT
	Sp-ply-556probe^1^	FAM-ACCCCAGCAATTCAAGTGTTCGCG-BHQ1
	Sp-lytA330-probe^1^	Cy5-TTTGCCGAAAACGCTTGATACAGGG-BHQ3
	Sp-lytA-CDCprobe^2^	FAM-GCCGAAAACGCTTGATACAGGGAG-BHQ1
	Sp-lytA-probe^2^	FAM-TTTGCCGAAAACGCTTGATACAGGG-TAMRA
	Sp-Spike-qPCR^3^	ACGCAATCTAGCAGATGAAGCTATCATGGCGACGTGTTTCATGCAGATATATCGGTAAACGATTTGTTGGCTAAG
	Sp-spike-probe^3^	HEX-CATGGCGACGTGTTTCATGCAGATA-BHQ1
Clinical CST	CST_01-M13F	GTAAAACGACGGCCAGCATTCGCATATCGTTTTTG
	CST_03-M13F	GTAAAACGACGGCCAGCATTCGCACATCGTCTTTG
	M13wzh1553F	GTAAAACGACGGCCAGACCATTGTCTCTACCTCTCAC
	M13wzh1553Fst3	GTAAAACGACGGCCAGATGATTGTGTCTACTTCGCAT
	M13wzh1747R	CAGGAAACAGCTATGACATCAAGGCATAACGACTATCA
	CST_01-M13R	CAGGAAACAGCTATGACCTGAGCTCTTTTTTTCATGA
	CST_04-M13R	CAGGAAACAGCTATGACCCGAGCTCTCTTTTTCATGA
	M13F	GTAAAACGACGGCCAG
	M13R	CAGGAAACAGCTATGAC

^1^used in the general study [8,35].

^2^used in the comparison sample set [40].

^3^DNA sequence used for internal control of the qPCR.

For comparison, the qPCR was performed by amplification of a segment of the *lytA* gene only. The primer and probe sequences were different from those applied in the sample set of the general study (Table 1). The 25 μl PCR mix contained 1x TaqMan® Universal PCR Master Mix, No AmpErase® UNG, 0.2 μM of each primer, 0.2 μM probe and 8 μl of the extracted DNA. Thermal cycling was performed in a 7500 Fast Dx qPCR Instrument (Applied Biosystems® Foster City, CA, USA) with the following cycling conditions: 2 min 50°C, 10 min 95°C and 50 cycles of 15 sec 95°C and 1 min 60°C. The standard curve consisted of a 10-fold dilution series of genomic pneumococcal DNA extracted by the Qiagen Genomic-tip 20/G Kit (Qiagen) and quantified by a spectrophotometer (Nanodrop ND-1000).

#### Capsular sequence typing (CST)

In CST a partial segment of the capsular gene *wzh* of the pneumococcus is amplified and subsequently sequenced [27]. Based on this sequence the capsular genotype is assigned, designating the serotype. In this study, the CST protocol was adapted to be performed directly on blood plasma samples and serum. To enhance sensitivity, the gene segment used in CST was amplified and sequenced in two overlapping segments which we subsequently assembled to create a single sequence. The primers were designed with 5′-M13-tails to facilitate DNA sequencing with a single M13 primer set (Table 1). To amplify the two gene segments 25 μl PCR mixtures containing Hotstartaq mix (Qiagen), 10 μM of each primer and 5 μl Sephadex purified DNA template were used. The PCR reaction was performed as follows: 15 min 95°C, 10 cycles of 20 sec 95°C, 30 sec 61-51°C and 30 sec 72°C followed by 35 cycles of 20 sec 95°C and 1 min 72°C, followed by 7 min 72°C. PCR products were purified using ExoSAP-IT (GE Healthcare Life Sciences) according to the manufacturer’s instructions. One μl aliquots of the purified PCR products were used in sequence reactions with M13 forward and reverse primers using Big Dye Terminator technology (Applied Biosystems) on an AB 3730 genetic analyser. Data analysis for CST was performed using Bionumerics version 7.0 (Applied Maths, Sint-Martens-Latem, Belgium). All sequences of the CST were assembled, edited, trimmed and assigned a capsular type. A capsular type is a composite assignment; the first part of the assignment is based on the phenotype assessed by conventional serotyping and the second part of the assignment is the consecutive number of the capsular type belonging to the same serotype. As an example, CT09V-01 designated the first variant in *wzh* sequence of an isolate serotyped as 9V. The CST database is publicly available through www.rivm.nl/mpf/spn/cst.

### Inhibition multiplex immunoassay (IMIA) for polysaccharide detection in urine

Urine samples were thawed and 2 ml urine was added to 2 ml absolute ethanol and incubated at 4°C overnight. Samples were centrifuged for 15 min at 3200xg, supernatant was discarded and the pellet was resolved in 100 μl PBS (pH 7.2) and heated for 10 min at 95°C. After the samples were centrifuged for 15 min at 2000xg the supernatant was stored at −20°C until use in the IMIA.

The coupling of the polysaccharides to carboxylated microspheres (Bio-Rad Laboratories, Hercules, CA, USA) was performed as previously described [30,33]. Briefly, purified capsular polysaccharides were conjugated to Poly-L-Lysine. The conjugates were coupled to carboxylated microspheres. All capsular polysaccharides were obtained from the American Type Culture Collection (ATCC, Manassas, VA, USA) except for polysaccharide 6A which was kindly provided by Pfizer Inc. (New York, NY, USA). Type and factor rabbit sera were obtained from Statens Serum Institut in Denmark and were purified using Melon Gel IgG Purification Kit (Thermo Fisher Scientific Inc., Rockford, IL USA) according to manufacturer’s protocol. The antisera against 14 serotypes were pooled and incubated overnight at 4°C in adsorbent buffer containing 15 μg/ml CWPS Multi and 5% antibody depleted human serum (ADHS, Valley Biomedical, Winchester, VA) in PBS (pH 7.2). The mixture contained antisera in the following dilutions: serotype 9V, 1:300; serotypes 6A, 6B, 8, 18C, 23F, 1:1,000; serotypes 3, 4, 1:1,600; serotype 7F, 1:2,000; serotype 19A, 1:5,000; serotype 19F, 1:8,000; serotype 1, 1:10,000; serotype 14, 1:21,600 and serotype 5, 1:150,000.

An aliquot of 25 μl of urine samples was incubated in duplicate for 1 h at room temperature with 25 μl of the pooled antiserum on a plate shaker. Subsequently, per well 4000 beads of each serotype specific microsphere set were added and incubated for 1 h at room temperature on a plate shaker. After the microspheres were washed using PBS-Tween, the beads were incubated for 20 min at room temperature with 100 μl of a 1/200 dilution of R-phycoerythrin-conjugated anti-rabbit IgG (Southern Biotechnology, Birmingham, AL, U.S.A.). After another wash of the microspheres, 125 μl PBS-Tween was added and microspheres were analysed using the Bio-Plex 100 (Bio-Rad, Hertfordshire, United Kingdom). Detection of polysaccharide in urine samples was considered positive using a 30% positivity cut-off value from the mean median fluorescent intensity (MFI) of duplicate pooled samples without competitive inhibition. The intra-run reproducibility was calculated as the coefficient of variation (CV) of replicates and a CV of 10% was considered to be acceptable. Polysaccharides of serotypes 1, 3, 4, 5, 6A, 6B, 7F, 8, 9V, 14, 18C, 19A, 19F and 23F were pooled and prepared in 10-fold dilutions from 1000 ng/ml to 0.01 ng/ml in PBS and were used as reference range in the assay.

### Multiplex Immunoassay (MIA) for assessment of longitudinal antibody concentrations

In two consecutive serum samples, an early (day 0–3) and late (day 11–100) sample, pneumococcal antibody concentrations were measured against 14 different serotypes (1, 3, 4, 8, 9N, 12F, 14, 19F, 23F, 6B, 7F, 18C, 19A, and 9V) using Luminex XMAP technology. Antibody concentrations were calculated by interpolation from a 5-parameter logistic standard curve [36,37], plotting the MFI against the polysaccharide concentration (Luminex software). A positive antibody response was defined as a serotype-specific twofold increase in antibody concentrations between the two samples. This method is described in more detail elsewhere [9].

### Statistical analysis

Statistical tests were performed using Graphpad Quickcalc software. Agreement between two tests was assessed by Cohen’s kappa (κ) statistic, with values indicating poor (0.00-0.20), fair (0.21 to 0.40), moderate (0.41-0.60), good (0.61-0.80) and excellent agreement (0.81-1.00).

## Results

### Optimization and implementation of the qPCR, CST and IMIA methods

Quantitative detection using qPCR was based on two target genes, *ply* and *lytA*. Overall, the *ply* target yielded amplification signals in more samples as compared to the *lytA* target indicating possible false positivity. Therefore, a sample was considered positive for pneumococci if at least one of the duplicate samples yielded a fluorescent signal for *lytA*. Based on serial dilutions of quantified PCR products, the detection limit was estimated to be 1.6 gene copies per microliter extracted DNA per PCR. The minimal bacterial load, determined using colony forming units (CFU) dilution series, that could be detected in the qPCR was 75 CFU per milliliter blood. Specificity of the assay was high as genomic DNA of closely related Streptococci, including *S. oralis, S. gordonii, S. parasanguinis, S. sanguinis* and *S. mitis* and *S. mutans* did not yield PCR products. To determine which serotypes can be detected using the primers amplifying the two overlapping segments in CST, lysates of pneumococcal isolates of the 45 serotypes most prevalent among IPD patients in The Netherlands were tested. Only the isolates with serotypes 10A, 22A, 23A, 24F, 25, 38 and 35B yielded non-specific or no PCR products in 1 of the two PCRs. The minimum amount of DNA required to perform typing by CST was around 11 gene copies per PCR. Once the assay was optimized, a comparison set of serum samples from 19 pneumococcal CAP patients were analysed by CST. In the serum samples with a bacterial load of >1800 gene copies per ml serum, a serotype could be identified by CST (Table 2). The serotypes identified were serotype 3 (5x), 19A (2x), 7F (2x), 4 (2x) and serotype 8 (1x) and there was full concordance between CST on serum and Quellung reaction and/or multiplex PCR on blood culture isolates.

**Table 2:** **Serotypes and bacterial load assessed in the comparison set (n = 19 samples of bacteremic patients)**

**Copies/ml serum**	**Serotype by Quellung/multiplex PCR**	**Capsular type by CST on serum**
22319	4	04-01
16865	3	03-01
15933	19A	19A-01*
10243	19A	19A-01
9803	3	03-03
8260	3	03-01
5954	7 F	07 F-01
5767	8	08-01
5533	7 F	07 F-01
1961	4	04-0*
1852	3	03-0*
1804	3	03-03
670	NT	no seq.
656	8	no seq.
155	5	no seq.
123	1	no seq.
122	8	no seq.
101	9 V	no seq.
92	7 F	no seq.

*Sequence <506 bp.

No seq., no sequence obtained or not possible to determine the serotype.

Specificity of the IMIA was assessed with a mixture of polysaccharide coated microspheres and pooled antisera, spiked with polysaccharides from a single serotype for inhibition. This method enabled the identification of all serotypes with negligible cross reactivity. The minimal required concentration of polysaccharides for the assay was assessed using spiked polysaccharides in urine and was between 1 and 10 ng/ml urine.

### Comparison of qPCR, IMIA and MIA with the conventional methods

A total of 309 patients were enrolled in this study and in these patients the causative agent of CAP was investigated using conventional microbiological methods. For 211 (68%) patients, blood was available for detection of pneumococci by qPCR and CST was performed if the samples were positive in the qPCR. The IMIA was performed on urine of 223 (72%) of the enrolled CAP patients. The serologic antibody response was assessed on samples of 171 (55%) patients. In 64 (21%) patients the pneumococcus was identified as causative agent of CAP by at least one conventional microbiological method: blood culture (28/301, 9%), sputum culture (15/294, 5%) and/or BinaxNOW urinary antigen test (52/307, 17%) (Table 3). In total, 8% (16/211) of patients were positive for pneumococcal infection using the qPCR, 17% (39/223) of patients using the IMIA and 22% (37/171) using the MIA for serologic antibody response. The different assays respectively obtained sensitivity of 29% (13 samples were qPCR positive of the 45 available samples for qPCR of patients that were positive by any of the conventional methods, Table 3), 43% (IMIA; 20/47) and 40% (MIA; 19/48) of the pneumococcal pneumonia patients identified by conventional methods.

**Table 3:** **Contribution of the alternative methods to the diagnosis of pneumococcal CAP**

			**All conventional tests**		**Blood culture**			**BinaxNow**			**Sputum culture**		
		**Total**	**Pos N (%)**	**Neg N (%)**	**Pos N (%)**	**Neg N (%)**	**Nd N**	**Pos N (%)**	**Neg N (%)**	**Nd N**	**Pos N (%)**	**Neg N (%)**	**Nd N**
	Total	309	64	245	28	273	8	52	255	2	15	279	15
qPCR	Pos	16	13 (29)	3 (02)	9 (45)	4 (2)	3	10 (29)	6 (3)	0	4 (33)	9 (5)	3
	Neg	195	32 (71)	163 (98)	11 (55)	180 (98)	4	24 (71)	169 (97)	2	8 (67)	176 (95)	11
	Nd	98	19	79	8	89	1	18	80	0	3	94	1
IMIA	Pos	39	20 (43)	19 (11)	11 (55)	27 (14)	1	16 (41)	23 (13)	0	3 (38)	32 (16)	4
	Neg	184	27 (57)	157 (89)	9 (45)	171 (86)	4	23 (59)	161 (86)	0	5 (62)	172 (84)	0
	Nd	86	17	69	8	75	3	13	71	2	7	75	4
MIA	Pos	37	19 (40)	18 (15)	9 (43)	26 (10)	2	13 (36)	23 (9)	1	6 (40)	23 (9)	8
	Neg	134	29 (60)	105 (85)	12 (57)	230 (90)	4	23 (64)	222 (91)	1	9 (60)	232 (91)	5
	Nd	138	16	125	7	17	2	16	10	0	0	24	2

Pos: positive, Neg: negative, Nd: not done/not available.

An additional 36 patients (56% increase, 100 patients in total), who were not recognized as pneumococcal CAP patients using the conventional methods, had a positive result in any of the three alternative methods. Four of these patients had a positive result in ≥2 of the alternative test methods. For two patients, samples had a positive result by CST and IMIA, and for two patients, samples had a positive result by MIA and IMIA (data not shown). The MIA and IMIA resulted in similar percentages of positive samples when conventional tests were negative (MIA 15%, 18/123; IMIA 11%, 19/176). The qPCR performed on blood samples detected lower number of additional positives (2%, 3/166) (Table 3).

Comparing the results from the assays that make use of the same clinical materials, in 20 patients for whom blood culture was positive and material for qPCR was available, 45% (9/20) yielded a positive qPCR (Table 3). In contrast, in blood culture negatives, 2% (4/184) of the samples were positive by qPCR. Antibody responses measured by MIA provided a similar number of positive results compared to blood culture. 43% (9/21) Of the patients that were found positive for pneumococci using blood culture, showed an antibody response using MIA. Of BinaxNOW positive patients, 41% (16/39) had a positive result in IMIA as well. An additional 13% (23/184) of BinaxNOW negative patients were tested positive by IMIA. Notably, if in a patient the pneumococcal infection was provisionally diagnosed by sputum culture (n = 15), the alternative tests yielded the lowest number of positive tests. The IMIA yielded the largest number of positives if sputum cultures were negative (16% (32/204). Specificity of the assays were 84% (32/204; IMIA vs. Sputum culture) and up to 98% (4/184; qPCR vs. blood culture). The cohens κ of agreement between assays ranged from 0.083 (poor; Sputum culture vs. IMIA) and 0.507 (moderate; Blood culture vs. qPCR) and was on average 0.288 (fair agreement).

### Serotypes deduced by alternative methods

The alternative methods enabled the identification of the serotypes in samples from 25% of all CAP patients (68 of 278 samples assessed using any of the alternative methods). In comparison, Quellung could have been performed on the infecting pneumococcus of 14% of patients (43 of 308 patients were found positive using blood or sputum culture). There was good concordance between the results obtained in the three methods. Serotypes 3 and 8 were the most frequently identified pneumococcal serotypes, each found in 15 patients (22%) (Table 4). Serotype 1, 9 V and 7 F were detected in samples from 7 (10%), 6 (9%) and 3 (4%) patients, respectively. In samples from 16 of the 68 patients (24%), two or more methods yielded identical serotypes. Once, the identical serotype was detected in all three different methods. In three cases, different methods yielded conflicting results. However, in these cases ambiguous IMIA results were included with weak signals for multiple serotypes.

**Table 4:** **Distribution of serotypes in this study detected by CST, MIA and IMIA**

**Serotypes**	**Frequency** ^**1**^	**Percent**	**CST (number tested: n = 16)**	**MIA (number tested: n = 171)**	**IMIA (number tested: n = 223)**
3	15	22	3	6	11
8	15	22	1	10	8
1	7	10	4	2	6
9V	6	9	1	2	4
7F	3	4	0	4	0
19A	2	3	0	2	1
4	2	3	1	3	0
6B	2	3	1	2	1
12F	1	1	0	1	NA
14	1	1	0	3	0
18C	1	1	0	1	0
23F	1	1	0	0	1
6A	1	1	0	NA	1
9N	1	1	0	1	NA
Ambiguous^2^	10	15			6
Total	68	100	11	37	39

^1^Number of the patients infected with a given serotype detected by any of the methods.

^2^No serotype could be determined because of cross reactivity with multiple serotypes in the IMIA or ambiguous results between methods.

NA. not applicable, serotype could not be assessed using the method.

## Discussion

In this study, samples from patients with CAP were used to evaluate the sensitivity of detection of *S. pneumoniae* with three different methods and to assess the added value for serotyping by non-culture methods. The advantage of the methods described in this study is that they can be used after antibiotic treatment and are time-saving, as culturing is not required. To the best of our knowledge, this is the first study that compares multiple assays used for detection and serotyping of pneumococci directly from clinical samples without the use of culture. The results of the three alternative methods revealed considerable overlap with the results of the conventional methods. Using the alternative methods, we were able to detect the pneumococcus in samples from 56% more patients compared to conventional methods, providing there were no false positive samples. Furthermore, of all patients with pneumonia, a pneumococcal serotype was determined in 25% using PCR/CST, IMIA and MIA as compared to 14% in which an isolate was available for serotyping using conventional methods

In general, the sensitivities of the individual tests described in this study are in line with similar assays developed by others. In our study, a number of samples were not available for each test. If only the patients for whom all tests were performed were included, sensitivity and specificity were higher compared to the numbers provided here. However, numbers were small and therefore we presented the data from the incomplete sampling.

In our study we enrolled patients with a pneumococcal CAP and non-pneumococcal CAP. The non-pneumococcal CAP patients account for the controls and are used to calculate specificity. However, it would have been valuable to include non-pneumonia patients for specificity calculations. Conventional tests (blood cultures, sputum cultures and BinaxNOW urinary antigen test) lack sensitivity and are therefore a far from ideal golden standard for specificity calculations. Positive results in the investigated assays for patients with negative results in the conventional tests should not be regarded as false-positive.

In a study by Abdeldaim et al., in CAP patients, the percentages (9%) of *lytA* PCR positive samples was comparable, but sensitivity of the *lytA* PCR compared to blood culture was slightly higher [38]. Cremers et al. showed a considerable higher number of qPCR positives (68%) in adults with blood-culture proven pneumococcal CAP [16]. In a study by Marchese et al., the sensitivity of the qPCR compared to blood culture was considerably higher, but this study included only pediatric patients [17]. In pediatric patients the blood culture positivity rate is considerably lower, which may be explained by lower bacterial load in children and small sample volumes of pediatric patients [39]. Also, differences in the amount of template DNA and number of PCR cycles could account for the differences in sensitivity between these studies. Also, primers used in these studies differed from our study. These factors may influence the sensitivity. The detection limit of the qPCR described here is comparable to the proposed CDC method, both <10 copies per PCR reaction [40]. In the comparison set as well as the general study, above a specific pneumococcal density threshold CST on clinical specimens from CAP patients was successful and accurate, despite differences in study region, cohort characteristics, clinical specimen, extraction method and qPCR conditions. The qPCR used in this study is based on the previously published qPCRs, described by McAvin et al. for *lytA* [8] and Greiner et al. for *ply* [35]. Other detection qPCR for pneumococci have been proposed, targeting these or other genes [40-42]. The *lytA* gene has been showed to be highly specific [40], whereas the *ply* gene is also carried by other flora normally present in the respiratory tract, such as *S. mitis and S. oralis*, and therefore may produce false positives in detection of pneumococci [40,42,43]. The *ply* target was amplified in more samples compared to the *lytA* target, indicating the non-specificity of the *ply* gene and indicating the correct choice of *lytA* as the primary target for the detection of pneumococci. The same was described in the study of Palmu et al. [42]. In our study, 69% of the blood samples with positive result in the qPCR could be assigned a serotype using CST. In the study by Marchese et al., molecular serotyping directly on blood was successful in 36 of the 46 (80%) samples from pediatric patients infected by pneumococci confirmed by culture and/or molecular test (real-time PCR amplification of both *lytA* and *cpsA* genes) [17]. In another study, in 73 of the 80 qPCR positive blood samples a serotype could be detected from patients of 0–16 years of age using specific PCRs to detect 21 different serotypes [18]. The CST has proven to be a valuable method to assess the serotype of an isolate in over 3000 isolates [27,28] (www.rivm.nl/mpf/spn/cst). Also, the method is quick and easy to perform. Because this method is based on a single gene in the capsular gene locus, some serotypes cannot be distinguished, eg. serotypes 18B and 18C. In general, one of the serotypes with corresponding sequence is not common in invasive pneumococcal disease, such as serotype 18B, but we are currently improving this assay to enable differentiation between those serotypes. Because the PCR target of the CST was divided in 2 segments to improve sensitivity, some serotypes could no longer be detected by this technique. However, the undetectable serotypes (serotypes 10A, 22A, 23A, 24F 25, 38, 35B) accounted for approximately 4% of IPD after vaccine introduction (data annual report of Dutch pneumococcal surveillance). Therefore, the increase in sensitivity caused by the amendment of the original assay outweighed the loss of the detection of these serotypes for the Dutch situation. However, in other parts of the world these serotypes may have increased after vaccine implementation [44].

The inhibition multiplex immunoassay (IMIA) described here detects the 13 serotypes included in PCV13 and serotype 8, because of the high incidence of invasive disease caused by this serotype. A similar assay was described by Findlow et al. with comparable sensitivity and specificity [33]. Main differences were the use of CPS multi as a sorbent and the use of rabbit antisera instead of a reference serum with a limited supply. In a different study using the Luminex platform, a serotype detection system was developed in an antigen capture approach. This assay uses monoclonal antibodies coupled to the microspheres and polysaccharide is detected in urine with the use of polyclonal anti-polysaccharide antibodies [10]. The authors showed a high specificity, which was supposed to be a benefit from the use of monoclonal antibodies. In an immune-inhibition approach like our method, commercially available antiserum is used. The use of commercially available antisera provides the possibility to expand the assay easily to detect more serotypes. Specificity and sensitivity in the study by Pride et al. were calculated using samples from patients of which the serotype of the pneumococcus was known to be a serotype that can be detected by the used assay, in contrast to our study. Therefore the accuracy of the tests cannot be compared correctly. In our study no samples were available for validation of the urinary assays such as the comparison set of blood samples. Although this is a drawback of the study, the good concordance of serotypes between the different assays indicates the correct assignment of serotypes.

Serotype distribution in our study was comparable to the serotype prevalence recorded by our national surveillance system on IPD [45]. In other studies targeting CAP patients, serotype distribution was also similar, although serotype 19A is increasingly detected in studies conducted a few years after introduction of pneumococcal conjugate vaccine [46]. Furthermore, distribution of serotypes is dependent on geography. For instance, in contrast to our study, serotype 5 was detected in CAP studies in the USA and UK [46,47]. Serotype 5 is mainly found in outbreaks in Europe [48]. Of note, the serotypes that are detected in surveillance studies will depend on the methods used. For example, urinary antigen assays are often detecting only 13 or 14 serotypes [10,32]. PCRs performed on blood samples to assign a serotype detect more serotypes, but this is also limited [17,18,46].

## Conclusion

In this study we assessed the use of three additional methods to identify and concurrently serotype a pneumococcus infecting patients hospitalized with CAP. Using these methods, we detected the pneumococcus in samples from 56% more patients compared to those found based on conventional methods. Also, we were able to assign a serotype to the infecting pneumococcus from samples of 25% of the patients using any of the three methods, compared to 14% of isolates obtained by blood and sputum culture suitable to determine the serotype by Quellung. This study indicates the usefulness of additional molecular methods to conventional laboratory methods for the detection of pneumococcal pneumonia. Moreover, detection of pneumococcal serotypes directly from clinical samples may improve the surveillance of the pneumococcal vaccine effectiveness.
